# Mitochondrion-mediated iron accumulation promotes carcinogenesis and Warburg effect through reactive oxygen species in osteosarcoma

**DOI:** 10.1186/s12935-020-01494-3

**Published:** 2020-08-18

**Authors:** Shuo Ni, Yanbin Kuang, Yin Yuan, Baoqing Yu

**Affiliations:** 1 Department of Orthopedics, Shanghai Pudong Hospital, Fudan University Pudong Medical Center, 2800 Gongwei Road, Pudong, Shanghai, 201399 China; 2 Department of Respiratory Medicine, School of Medicine, Ren Ji Hospital, Shanghai Jiao Tong University, Shanghai, 200127 China; 3 State Key Laboratory for Diagnosis and Treatment of Infectious Diseases, The First Affiliated Hospital, School of Medicine, Zhejiang University, Hangzhou, 310003 China

**Keywords:** Iron, Mitoferrin, Warburg, Reactive oxygen species, Osteosarcoma

## Abstract

**Background:**

Iron metabolism disorder is closely associated with several malignant tumors, however the mechanisms underlying iron and the carcinogenesis in osteosarcoma are not yet well understood.

**Methods:**

Cell proliferation ability of osteosarcoma cell lines was measured by CCK-8, EdU incorporation and colony formation assays. Cell cycle analysis was detected by flow cytometry. The carcinogenesis of osteosarcoma was measured by soft-agar formation, trans-well and Wound healing-scratch assay. Warburg effect was detected by Seahorse respirometry assays. Reactive oxygen species (ROS) level was measured by Dichlorodihydrofluorescein diacetate (DCFH-DA) fluorescent probes. Western blotting was used to measure the expression of mitoferrin 1 (SLC25A37) and mitoferrin 2 (SLC25A28). Iron level in vitro and vivo was detected by iron assay kit. RNAi stable cell lines was generated using shRNA.

**Results:**

Iron promoted proliferation, carcinogenesis and Warburg effect of osteosarcoma cells. Iron-induced reactive oxygen species (ROS) played an important role in these processes. Iron accumulated more in mitochondrion than in cytoplasm, suggesting mitochondrion-mediated iron accumulation was involved in the development of osteosarcoma. Moreover, iron upregulated the expression of mitoferrin 1 (SLC25A37) and mitoferrin 2 (SLC25A28). Knock-down of mitoferrin 1 (SLC25A37) and mitoferrin 2 (SLC25A28) decreased the production of ROS. In addition, iron increased the expression of Warburg key enzymes HK2 and Glut1, and affected AMPK/mTORC1 signaling axis.

**Conclusions:**

Mitochondrion-mediated iron accumulation promotes carcinogenesis and Warburg effect of osteosarcoma cells. Meanwhile, iron deprivation might be a novel effective strategy in the treatment of osteosarcoma.

## Background

Iron metabolism is of central importance to numerous biological processes, including DNA replication, cell-cycle progression, electron transport chain and microsomal electron transport proteins, as well as the production of iron-sulfur proteins [1, 2]. Iron is absorbed from the duodenum by divalent metal transporter 1 (DMT1) and then transported by transferrin, which is regulated by hepcidin and cellular iron exporter ferroportin (FPN1) [3, 4]. Notably, mitochondrion is the main consumer of iron in cells for its function of synthesis or assemble many important proteins such as heme and iron sulfur clusters [5, 6]. Mitoferrin 1 (SLC25A37) and mitoferrin 2 (SLC25A28) are the main iron importers in mitochondrion, which controlled the iron homeostasis by transferring the ferrous iron to the mitochondrial matrix [7, 8]. Accumulating evidence indicates that mitochondrial iron accumulation is associated with human disease such as pulmonary and cardiovascular disorders [9–11]. However, little is known about the role of mitochondrion-mediated iron accumulation in the initiation and development of malignant tumors.

Several large epidemiological studies have demonstrated that iron contributes to the occurrence and progression of esophageal cancer, colorectal cancer, liver cancer, and lung cancer [12–15]. Dietary intake of high-iron is associated with increased colorectal cancer risks [16]. Moreover, iron overload promotes carcinogenesis and tumorigenesis in colorectal and lung cancer [17–19]. Some studies have demonstrated that iron could induce reactive oxygen species (ROS) and promote carcinogenesis [20]. Moreover, some studies also demonstrate that mitochondrion-mediated iron-dependent ROS accumulation promotes pancreatic tumorigenesis in mice [21]. Some iron chelator such as deferoxamine (DFO), Di-2-pyridylketone-4,4-dimethyl-3-thiosemicarbazone (Dp44mT) and ciclopirox olamine (CPX) have been showed with anti-cancer effects recently [18, 22, 23].

As a hallmark of cancer cells, Warburg effect is associated with high level of glycolysis even when cancer cells are under aerobic conditions [24]. Several studies have demonstrated that reactive oxygen species (ROS) regulate Warburg effect [21, 24–26]. In the present study, we investigated the association among iron, ROS and Warburg effects in carcinogenesis of osteosarcoma. Meanwhile, our work reveals that iron deprivation might be a novel effective strategy in the treatment of osteosarcoma.

## Methods and materials

### Cell lines and reagents

Human osteosarcoma cell line SAOS-2 and U2OS were obtained from American Type Culture Collection (ATCC, USA). Both cell lines were cultured in RPMI 1640 medium (Gibco, Gaithersburg, MD, USA) with 10% fetal bovine serum (FBS, Invitrogen, Carlsbad, CA, USA) in a concentration of 5% CO_2_ and 37 °C incubator. Ferric ammonium citrate (FAC) and Deferoxamine (DFO) were purchased from Sigma (St. Louis, MO, USA).

### Mouse xenograft tumor model

The animal experiments in this study was approved by the Animal Ethics Committee of Zhejiang University School of Medicine and in accordance with the National Institutes of Health (NIH) Guide for the animal treatments of Laboratory Animals. Twelve BALB/c nude mice were randomly divided into 2 groups. A number of 2 * 10^6^ SAOS-2 cells resuspended in 200 µl FBS-free RPMI 1640 medium was injected subcutaneously into the arms of nude mice (6-week-old, female). Tumor volumes were measured every 2 days. Deferoxamine (DFO) 16 mg/kg or normal saline (NS) was injected intraperitoneally for 2 weeks. Deferoxamine (DFO) was purchased from Sigma (St. Louis, MO, USA). After 2 weeks, all animals were sacrificed.

### Cell viability assay and cell cycle analysis

Cell counting Kit-8 (CCK-8; Beyotime, Shanghai, China) was obtained from Beyotime and be used to detect the viability of two cell line according to the manufactories’ protocol. FAC (100 μM), DFO ((100 μM)) and PBS were added to different groups respectively. Then the 96-well plate was assessed at 450 nm 24 h, 48 h and 72 h after incubation. For the cell cycle assay, a total of 1 × 10^6^ cells nearly 80% confluent from 6-well plate were collected and fixed in 70% ethanol for 24 h at 4 °C like previously reported [27]. Cells were then washed in Phosphate Buffer Saline (PBS) and stained with propidium iodide (PI) before being analyzed by flow cytometry (BD Biosciences). G1, S and G2/M phases were calculated by Modfit Software (Verity Software House, Inc.).

### Plate colony formation

Cells were collected and resuspended in RPMI 1640 medium containing 100 μM FAC and 100 μM DFO, respectively. Then, the cells were transferred to 6-well plates at the density of 500 cells per well and incubated for 14 days. The culture medium in different groups were replaced every 2 days until the end of this experiment. Finally, colonies were stained by 1% Giemsa stain solution (Solarbio, Beijing, China) for 30 min. All of the colonies were counted and quantified.

### EdU cell proliferation assay

Edu Cell Proliferation Kit (Beyotime, Shanghai, China) was used to detect the proliferation of two cell lines visually according to the manufactories’ protocol. After being stimulated by 100 μM FAC or DFO for 24 h respectively, cells were stained and captured by Olympus FSX100 microscope (Olympus, Tokyo, Japan).

### Soft-agar colony formation assay

Two percent agar solution was made in room temperature and sterilized by pressure cooker in 120 °C for 6 h, then it was stored in 4 °C in the refrigerator. Microwave oven was used to heat the solid agar until it was become liquid at 37 °C. Two percent of agar solution mixed with culture medium containing 100 μM FAC or 100 μM DFO was made respectively. Then the mixed medium was added into 24-well plate, and moved to 4 °C refrigerator immediately until it turns into solid. Then cell suspension containing each type of cell was added on to the top of solidified agar (500/50 μL). Thereafter, 2% agar solution containing each conditional medium (1:6 v/v) along with Matrigel (1:30 v/v). After 2 weeks of culture, colony numbers were counted.

### Trans-well assay

Trans-well chambers (Corning Costar, Cambridge, MA, USA) were used to detect the migration and invasion ability of cells. In brief, cells were cultured in FBS-free medium overnight. Then it was transferred to the upper chambers (10^5^ cells/well). Culture medium containing 100 μM was added into the lower chamber, followed by a 24-h incubation. The next day, cells on the upper surface (non-migrated cells) were gently removed by small sticks. The cells on the opposite surface (migrated cells) were counted and imaged. For the invasion assay, diluted (1:6) Matrigel (BD Bioscience, San Diego, CA, USA) was used to cover the surface of upper chamber before cells being planted on it. Other steps were the same as migration assay.

### Wound healing-scratch assay

SAOS-2 and U2OS cells were seeded in 12-well plates. After cells were confluence, wound area was made in cell monolayer by pipette tips. FBS-free RPMI 1640 medium containing 100 μM FAC or 100 μM DFO were added and incubated for 24 h. The wound closure was captured and the percentage of arear was evaluated by ImageJ software (USA).

### Seahorse XF24 respirometry assay

Seahorse Bioscience Extracellular Flux Analyzer (XF24, Seahorse Bioscience Inc., North Billerica, MA, USA) was used to detect the oxygen consuming rate (OCR), and extracellular acid rate (ECAR). Mito Stress Test Kit from Agilent was used according to the manufacturer’s protocol. Briefly, 1*10^4^ cells were seeded in the 24-well plate in conditional culture medium and incubated overnight. Then the cells were washed with XF media (1% FBS) then cultured in a CO_2_-free incubator at 37 °C for 2 h. ECAR and OCR measurements were performed. OCR and ECAR were measured in a typical 8 min cycle of mix (2 to 4 min), dwell (2 min), and measure (2 to 4 min).

### Mitochondrial extraction

Mitochondrial isolation and extraction were performed according to the manufacturers’ protocol. Mitochondria/Cytosol Fractionation Kits (ab65320, Abcam). Briefly, cells were harvested after culturing in different conditional medium for 24 h. A number of 5*10^7^ cells was centrifuged at 600×*g* for 5 min at 4 °C. Cells were resuspended with cytosol extraction buffer mix after washing with cold PBS. Then the cells were incubated 10 min and performed the task with grinder on ice.

### Iron assay

Iron assay was performed according to the manufacturers’ protocol of Iron Assay Kit (ab83366, Abcam) as previously described [21]. In brief, samples were incubated for 30 min at 25 °C, followed by an incubation of 60 min with iron probe at 25 °C. Then all the samples were moved to microplate reader.

### Generation of RNAi stable cell lines

Human SLC25A37 shRNA and human SLC25A28 shRNA sequences were designed by Biomics Biotech (Shanghai, China). Non-specific shRNA (NS) was used as control. Briefly, HEK293T cells were transfected by lentivirus-shRNA. After 48 h of incubation, culture medium containing lentivirus was used to infect SAOS-2 and U2OS cells lines. Lipofectamine 3000 was used in the transfection procedure. The transfection procedure was performed according to the manufacturers’ protocol of Lipofectamine 3000 (Invitrogen, Carlsbad, CA, USA). Puromycin (2 μg/ml) was used to screen stable cell lines. Knockdown of SLC25A37 and SLC25A28 were confirmed by qPCR and Western blot. The most effective sequence of SLC25A37 shRNA and human SLC25A28 shRNA are listed in Additional file 1: Table S2.

### ROS production detection

Dichlorodihydrofluorescein diacetate (DCFH-DA; Beyotime, Shanghai, People’s Republic of China) was used to detect ROS production according to the manufacturers’ protocol. Briefly, 5 * 10^5^ cells were planted in the 6-well plate in different conditional culture medium (100 μM FAC or 100 μM DFO) for 24 h. at the day of measurement, then the culture medium was removed. Next, FBS-free medium with DCFH-DA was added to the dish and then incubated for 20 min. The fluorescence intensity of cells was detected by microplate reader.

### TCGA database and analysis

The correlation of mitochondrion-related genes and Warburg genes was analyzed by GEPIA web tools (http://gepia.cancer-pku.cn/) based on the TCGA database.

### Western blot analysis

Cells were collected after stimulated with 100 μM FAC or 100 μM DFO for 24 h. Cellular proteins were extracted by RIPA lysis buffer containing protease and phosphatase inhibitors. SDS-PAGE was used to separate the proteins. After running process, gels were transferred to PVDF membranes and immersed in primary antibodies. The next day, membranes were incubated with secondary antibodies and be visualized by chemiluminescence detection kit (Beyotime). Slc25a28 antibody (ab90170, 1:100) was from Abcam. antibodies specific for SLC25A37/Mitoferrin1 (26469-1-AP, 1:100) and Glut1 (66290-1-Ig, 1:100) were purchased from Proteintech. Anti-phospho-AMPK (Thr172) antibody (#2535S, 1:100), Anti-AMPKα Antibody (#2532, 1:100), anti-p70-S6K (9202S, 1:100), anti-phospo-p70-S6K (Thr389) (9234S, 1:100), anti-Hexokinase 2 (2867S, 1:100), anti-phospho-4EBP1 (Thr70) (13396, 1:100) and anti-4EBP1 (9644s, 1:100) were from Cell Signaling Technology. Anti-PCNA (2586S, 1:100) was from Cell Signaling Technology. The anti-GAPDH antibody (BM1623, 1:1000), anti-β-actin antibody (BM0627, 1:1000), anti-α-tubulin antibody (BM1452, 1:1000), anti-rabbit IgG-HRP antibody (BA1054, 1:5000), and anti-mouse IgG-HRP antibody (BA1050, 1:5000) were purchased from Boster Biological Technology (Wuhan, China).

### RNA extraction and qRT-PCR

Cells treated with 100 μM FAC or 100 μM DFO for 24 h were collected for RNA extraction. RNeasy Mini Kit (Qiagen, Valencia, USA) was used to extract the RNA. Then it was reverse-transcribed into cDNA. The mRNA expression levels were assessed by qRT-PCR system (Applied Biosystems, Foster, CA, USA). The primers we used are listed in Additional file 1: Table S1.

### Statistical analysis

All experimental data was presented as the mean  ± SD (n  ≥  3). GraphPad Prism (version 7, GraphPad Software, La Jolla, CA, USA) was used to analyse the data. Student’s *t*-test was used between treated and control group. One-way ANOVA was used for multiple groups. LSD-t test was applied when data needed to be compared with control in multiple groups. p < 0.05 was considered to be significant.

## Results

### Iron chelator DFO decreases tumorigenicity in vivo

**Fig. 1:**
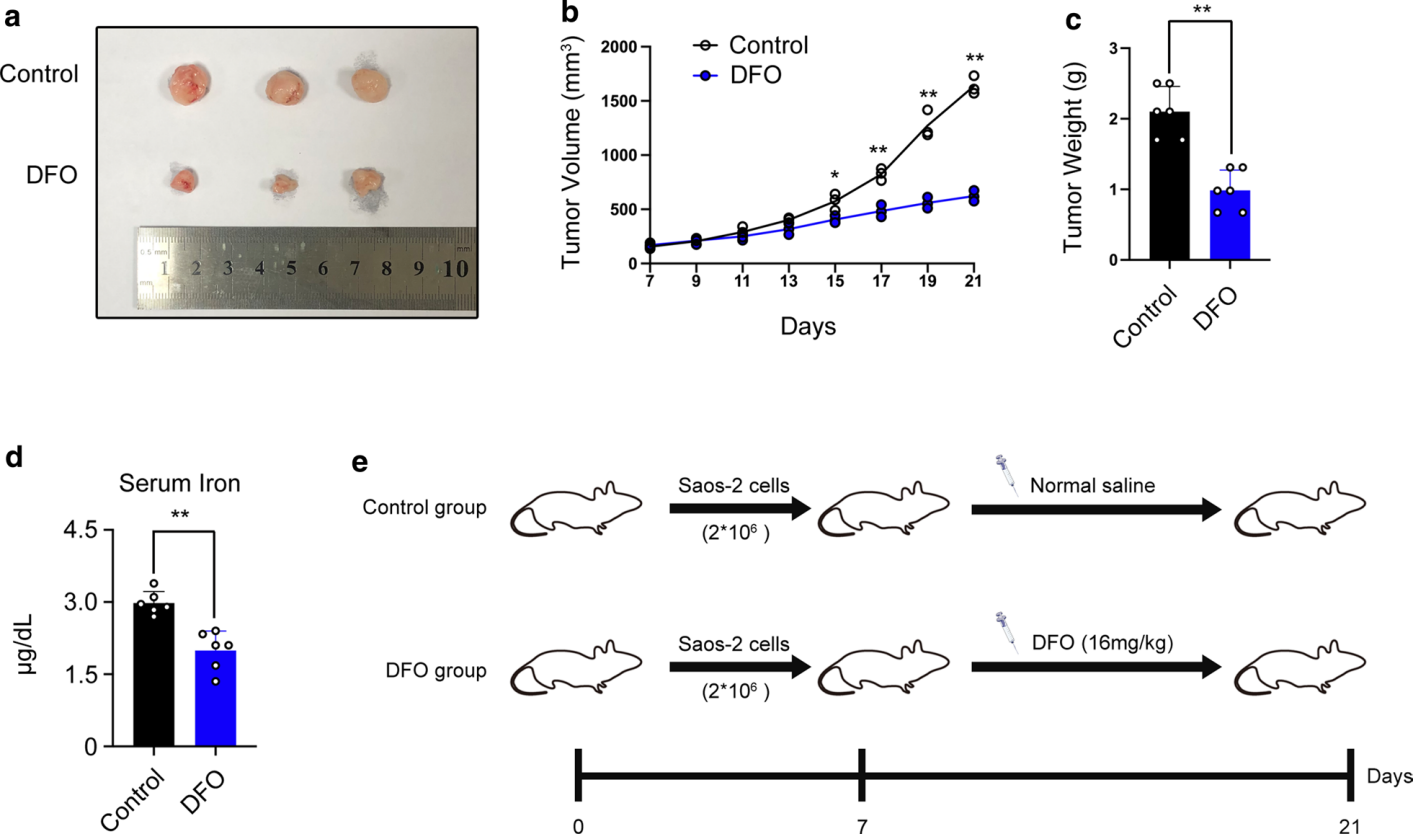
Deferoxamine (DFO) inhibited osteosarcoma development. **a** Representative images of tumors injected with 2 * 10^6^ SAOS-2 cells subcutaneously (n = 6). **b** Tumor volumes were measured by the indicated time (n = 6). **c** Tumor weight (n = 6). **d** Serum iron analyses (*p < 0.05, **p < 0.01, mean ± SD, n = 6). **e** Proposed scheme of animal experiment

Based on the epidemiological evidences that cancer patient links with an increased body iron storage 28, so we used Deferoxamine (DFO), a chelator used in clinic which could chelate iron efficiently, to explore whether iron was involved in the tumorigenesis of osteosarcoma in vivo (Fig. 1e). DFO group showed a smaller tumor volume and lower weight compared with control group (Fig. 1a–c). Moreover, DFO treatment led to a lower serum iron level when compared to control group (Fig. 1d). These results indicated that DFO could inhibit the development of osteosarcoma, which implied that iron maybe was involved in the tumorigenesis of osteosarcoma.

### Iron promotes osteosarcoma cell lines proliferation and affects cell cycle progression

**Fig. 2:**
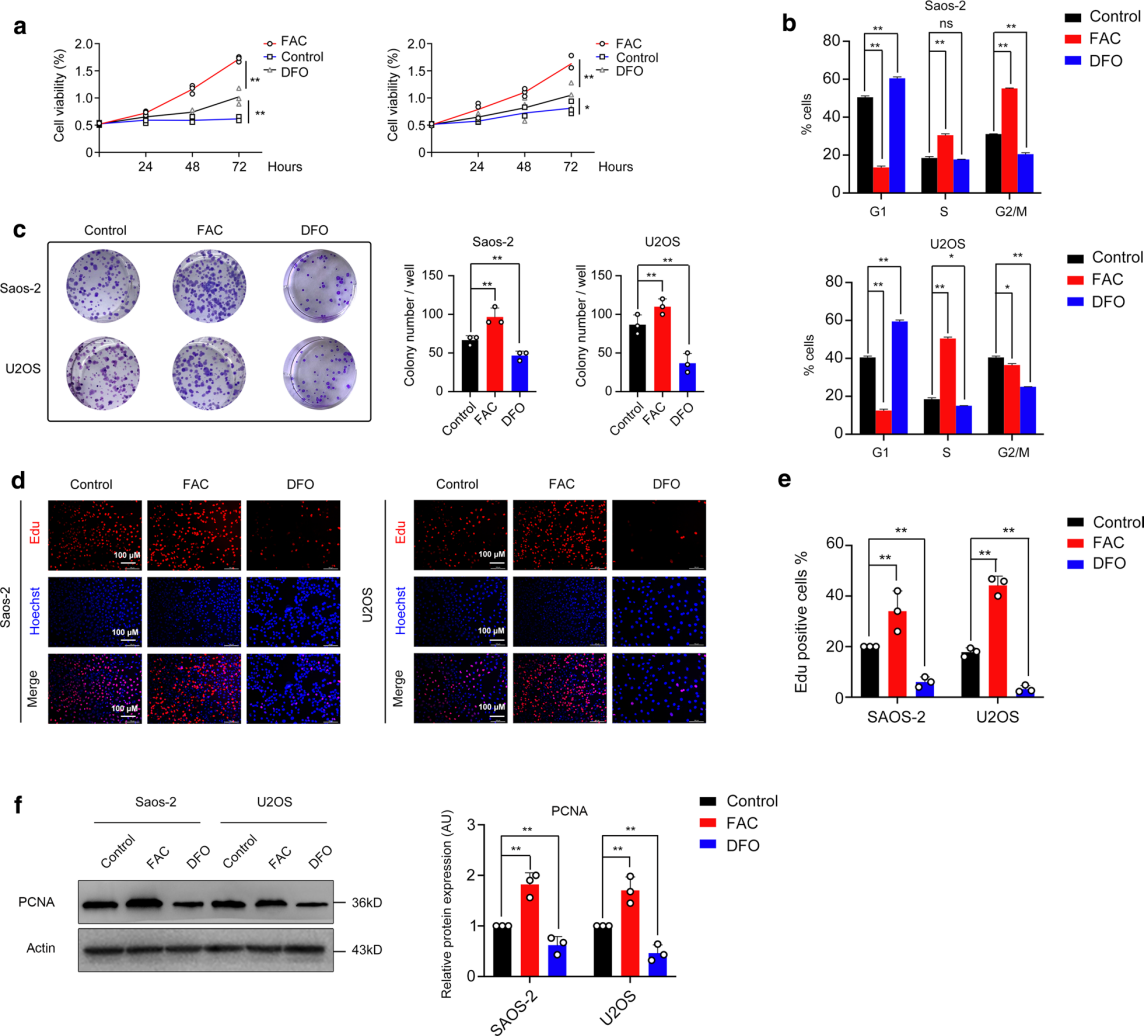
Iron promotes osteosarcoma cell lines proliferation and affects cell cycle progression. **a** Cell viability assay of SAOS-2 and U2OS cells treated with 100 µM FAC, 100 µM DFO or PBS for 0–72 h. **b** Cell cycle analysis of SAOS-2 and U2OS cells by flow cytometry. **c** Plate colony formation assay of each group. **d**, **e** Representative immunofluorescence images and percentages of EdU-positive cells. **f** Iron increased PCNA expression while DFO showed an inverse result (*p < 0.05, **p < 0.01, mean ± SD, n = 3)

Two osteosarcoma cell lines SAOS-2 and U2OS were treated with exogenous iron (ferric ammonium citrate, FAC), DFO or PBS at indicated times. We found FAC could increase the viability of both cell lines (Fig. 2a). Flow cytometry analysis revealed that FAC treatment increases the percentage of S phase and decreases the percentage of G1 phase. Whereas DFO treatment presented as na inverse results, which indicated that iron affects cell cycle progression (Fig. 2b). The colony formation assay showed an increase of colony number in FAC group, whereas DFO group showed a decrease of colony number compared to control group (Fig. 2c). The results of EdU cell proliferation assay showed an increase of cell percentage in cells treated with FAC (Fig. 2d, e). Together, these results demonstrated that the cell proliferative ability and cell cycle progression could be promoted by FAC while DFO inhibit these processes, indicating that iron could be a promoter in osteosarcoma cell lines proliferation. Proliferating cell nuclear antigen (PCNA) was reported to be an indicator in cell proliferation. We found that iron increased PCNA protein level while DFO showed an inverse result (Fig. 2f).

### Iron promotes osteosarcoma carcinogenesis and increases migration and invasion ability of both cells

**Fig. 3:**
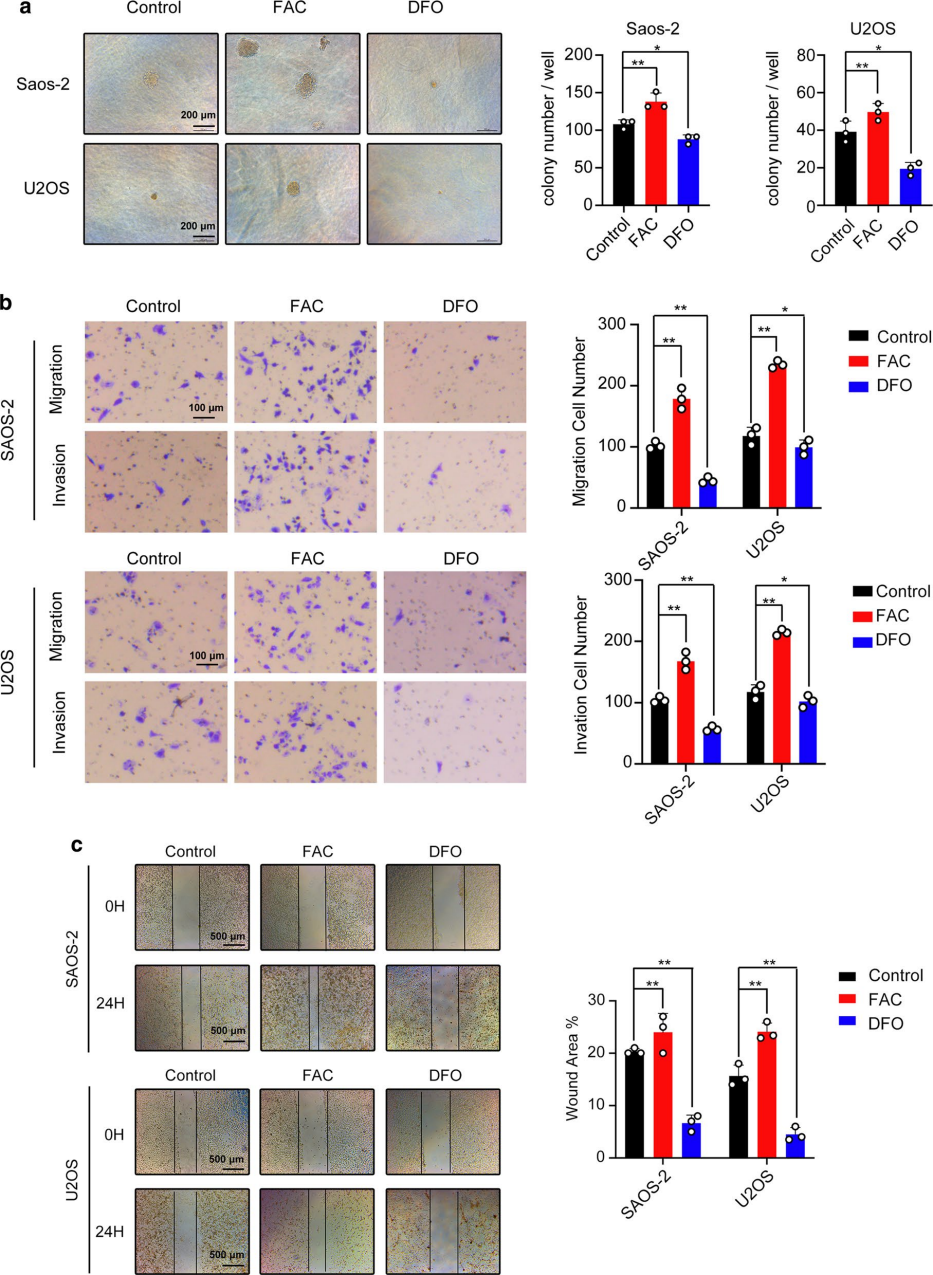
Iron promoted carcinogenesis, migration and invasion of osteosarcoma cell-lines SAOS-2 and U2OS. **a** Soft-agar colony formation assay showing ferric ammonium citrate (FAC) promoted the growth of osteosarcoma cells. **b** Ferric ammonium citrate (FAC) increased the migration and invasion of SAOS-2 and U2OS cells. **c** Ferric ammonium citrate (FAC) showed a smaller wound area after 24 h incubation (*p < 0.05, **p < 0.01, mean ± SD, n = 3)

The soft-agar colony formation assay was used to detect the carcinogenesis of osteosarcoma. Colony number of FAC-treated showed a significant increase compared to other groups (Fig. 3a). In the trans-well assay, FAC group showed more SAOS-2 and U2OS cells than any other groups (Fig. 3b). The wound healing assay, FAC group showed a smaller wound area (Fig. 3c). Collectively, these results indicated iron promotes carcinogenesis, invasion and migration ability of both osteosarcoma cells carcinogenesis, invasion and migration.

### Mitochondrion-mediated iron accumulation promotes Warburg effect

**Fig. 4:**
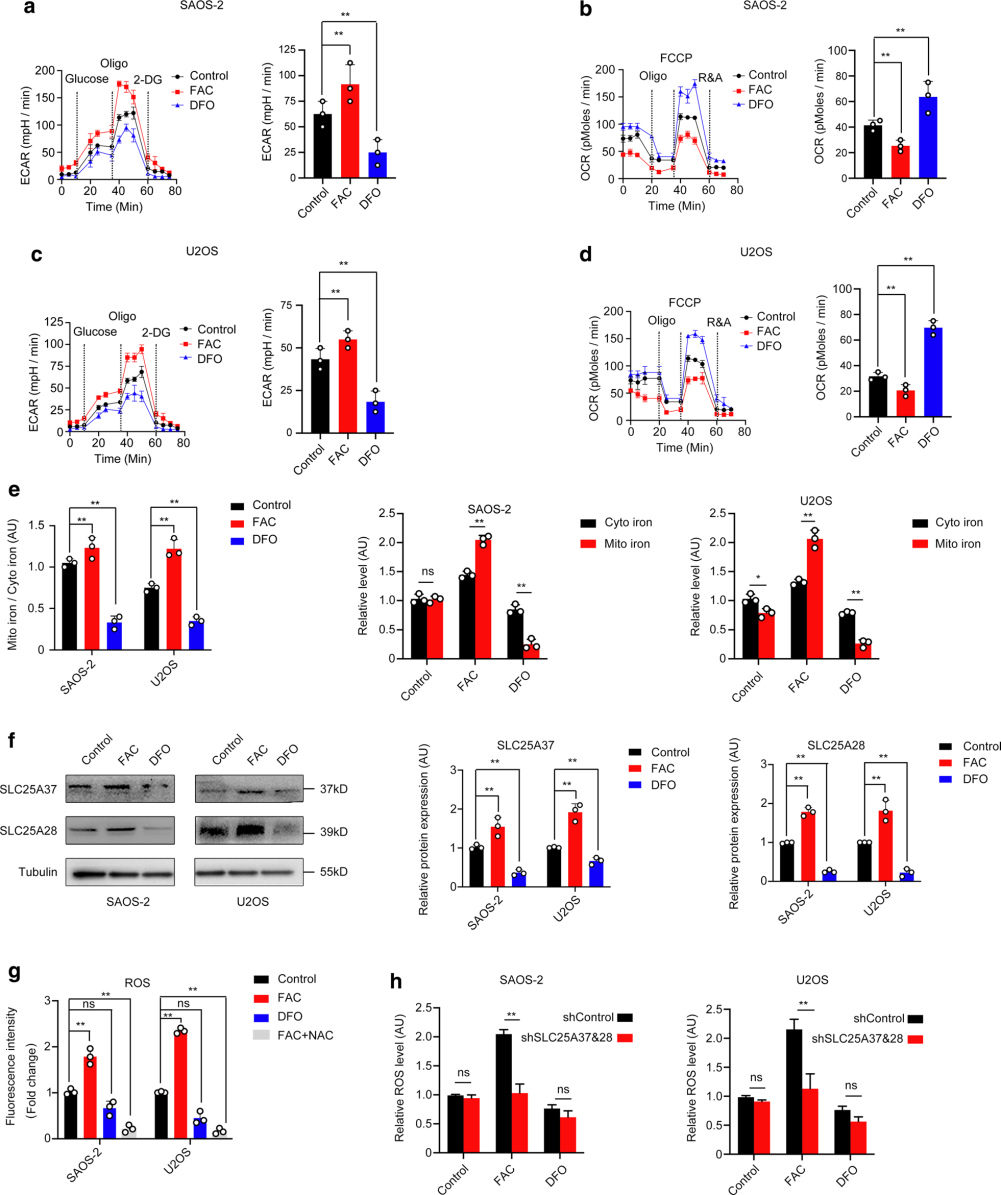
Mitochondrion-mediated iron accumulation promotes Warburg effect through reactive oxygen species. **a**–**d** Seahorse metabolic analysis of extracellular acidification (ECAR) and oxygen consumption rates (OCR) in both cell lines. **e** Mitochondrion iron accumulation was higher than that of cytoplasm. **f** Ferric ammonium citrate (FAC) increased mitoferrin 1 (SLC25A37) and mitoferrin 2 (SLC25A28) expression. **g** Ferric ammonium citrate (FAC) increased reactive oxygen species (ROS) production. **h** Depletion of mitoferrin 1 (SLC25A37) and mitoferrin 2 (SLC25A28) by shRNA rescued the ROS production by FAC (*p < 0.05, **p < 0.01, ns: no significant, mean ± SD, n = 3)

Warburg effect, as one of the hallmarks of cancer, presented as a metabolic disorder. Even in oxygen condition cancer cells acquire energy by aerobic glycolysis. Seahorse metabolic analyzer is used to detect the extracellular acidification (ECAR) and oxygen consumption rates (OCR). FAC-treated group showed a higher ECAR compared to that of control group, whereas DFO-treated group showed a reduced ECAR. OCR results in FAC group were significantly lower than other groups in both cell lines (Fig. 4a–d). We further wanted to investigate the places where iron accumulated in cells. Iron assay was used to detect the iron content in cytoplasm and mitochondrion according to the instruction of Iron Assay Kit. Mitochondrion to cytoplasm ratio indicated that iron was accumulated more in mitochondrion than that of cytoplasm (Fig. 4e). Next, mitochondrial iron importer proteins mitoferrin 1 (SLC25A37) and mitoferrin 2 (SLC25A28) were detected by western blot. We found FAC could increase the expression of mitoferrin 1 (SLC25A37) and mitoferrin 2 (SLC25A28) (Fig. 4f). Taken together, these results showed that iron promoted Warburg effect of osteosarcoma cells through the mitochondrion-mediated iron accumulation.

### Mitochondrion-mediated iron accumulation increases ROS production by mitoferrin

Studies have shown that iron could increase ROS production and promote carcinogenesis [20, 21]. We then explored ROS production in each group by DCFH-DA, and then found that FAC-treated group with a significant increase of ROS. A significant ROS inhibition was observed when ROS scavenger NAC (5 mM) was added into FAC culture medium for 2 h. Interestingly, DFO exhibited as a ROS scavenger (Fig. 4g). Next, we wanted to explore the underlying mechanisms of iron-mediated ROS production. Depletion of SLC25A37 and SLC25A28 by RNAi could significantly reduce ROS production when cells treated by FAC (Fig. 4h). The expression of shRNA was confirmed by western blot and qPCR Additional file 2: Fig. S1). Collectively, our results demonstrated that iron could increase ROS production by mitoferrin 1 (SLC25A37) and mitoferrin 2 (SLC25A28).

### SLC25A37 had a positive correlation with key genes of Warburg effect

**Fig. 5:**
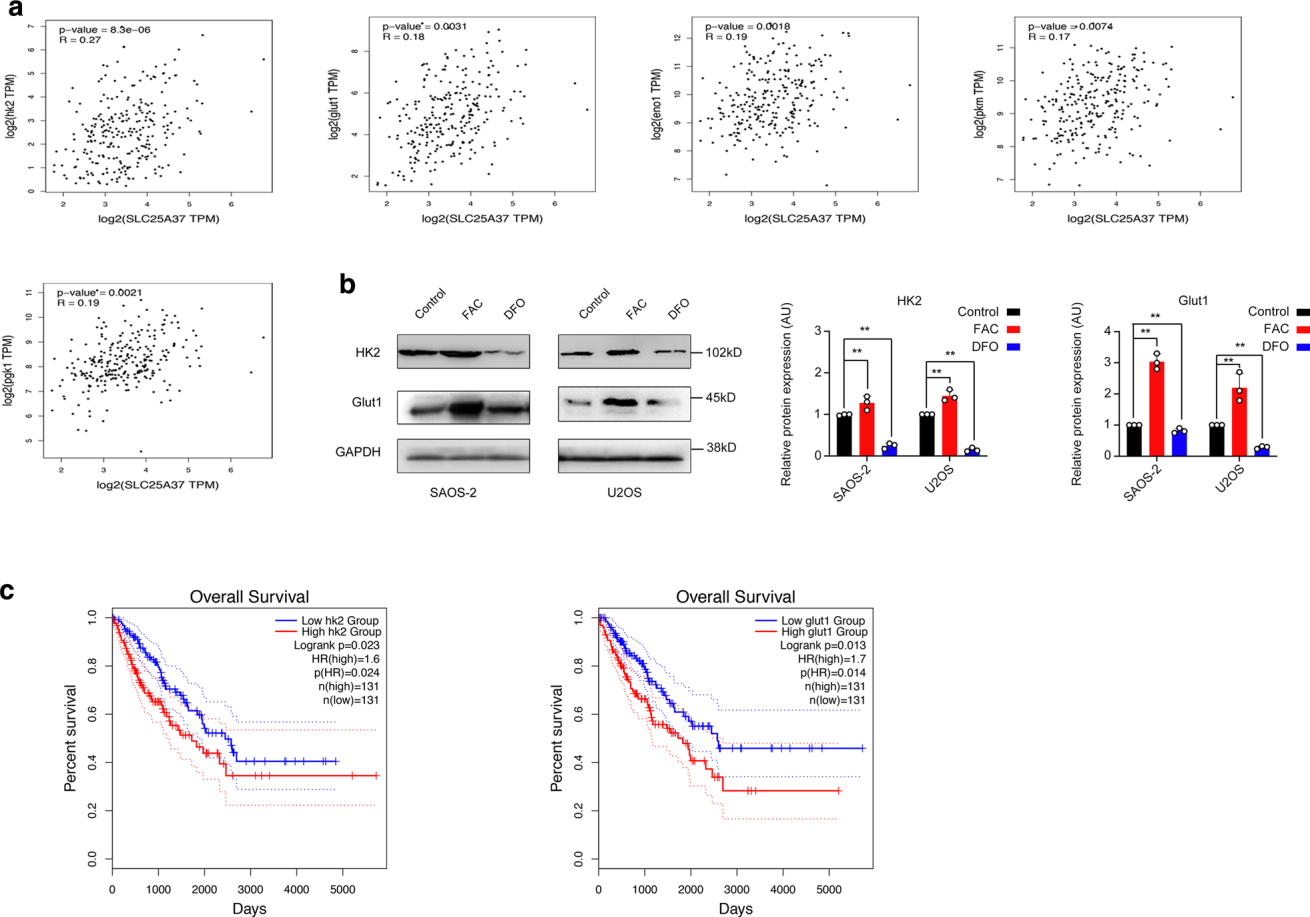
Mitoferrin genes co-expressed with Warburg key genes in sarcoma patients, high expression of HK2 and Glut1 were associated with poor prognosis in sarcoma patients. **a** Mitoferrin 1 (SLC25A37) had a positive correlation between Warburg key genes. **b** Ferric ammonium citrate (FAC) increased HK2 and Glut1 expression. **c** High expression of HK2 and Glut1 were associated with poor prognosis in sarcoma patients

Based on the results described above, GEPIA 2 online tools which based on TCGA database, was used to investigate the correlation of SLC25A37 and Warburg key genes including HK2, GLUT1, GAPDH, PGK1, ENO1, PKM and LDHA (http://gepia2.cancer-pku.cn/#correlation). GEPIA (Gene Expression Profiling Interactive Analysis) web server, which was based on tumor and normal samples from the TCGA and the GTEx databases, has been a valuable and highly cited resource for gene expression analysis since introduced in 2017. In addition, GEPIA2 has adopted new analysis techniques of gene signature quantification inspired by single-cell sequencing studies, and provides customized analysis where users can upload their own RNA-seq data and compare them with TCGA and GTEx samples. p < 0.05 was considered as statistically significant by using GEPIA2 web tools. HK2, GLUT1, PGK1, ENO1 and PKM were found positive correlation with SLC25A37 in sarcoma patients (Fig. 5a).

### Iron increased Warburg key protein HK2 and GLUT1 expression, which were associated with poor prognosis in osteosarcoma patients

According to the results we found by GEPIA 2 online tools and several studies reported, the expression of Warburg key proteins HK2 and Glut1 were significantly increased compared to control group, while DFO rescued the expression of HK2 and Glut1 (Fig. 5b). Further, we evaluated the correlations between those protein expressions and the survival of sarcoma patients. A number of 131 cases of sarcoma was analyzed and we found that HK2 and Glut1 high expression associated with poor prognosis in osteosarcoma patients, p = 0.023 and p = 0.013, respectively (Fig. 5c). Given the fact that we are unable to collect osteosarcoma specimen due to the lack of such patients in our hospital, so we didn’t perform those genes expression in clinic as other study reported [29].

### Iron may affect AMPK and subsequently promote mTORC1 activity

**Fig. 6:**
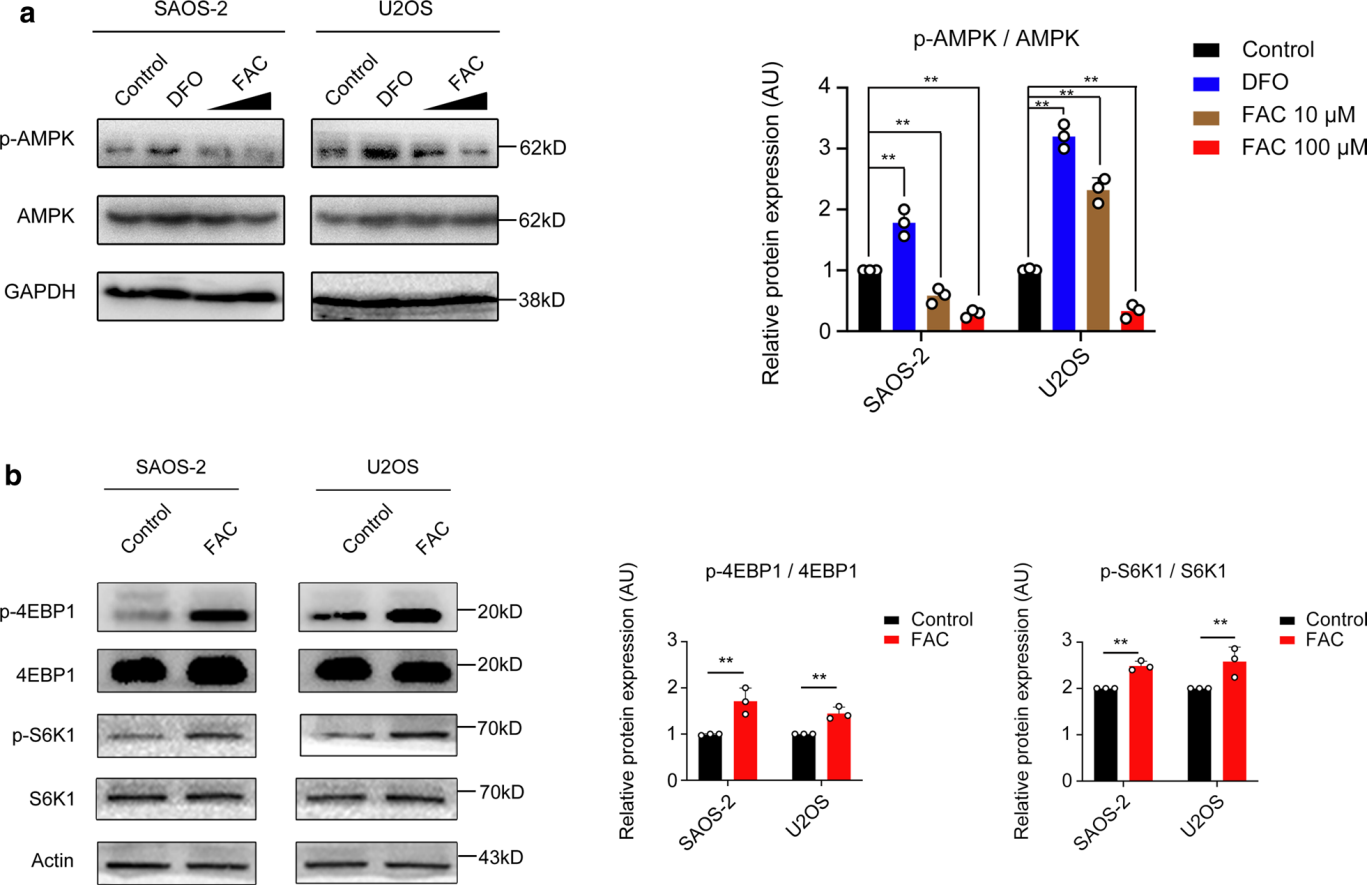
Iron-induced AMPK inhibition increased mTORC1 activity. Ferric ammonium citrate (FAC) inhibited expression of phospho-AMPK. Ferric ammonium citrate (FAC) increased phosphor-4EBP1 and phosphor-S6k1 activity

AMP-activated protein kinase (AMPK) worked as a regulator of cell metabolism and a sensor of energy, energy stress-induced activation of AMPK inhibits the activation of mTORC1, and mTORC1 is activated in the inverse pattern [30]. We next investigated whether this pathway is involved in iron-induced metabolism abnormal. The inhibition of p-AMPK and subsequently the activation of mTORC1 was observed (Fig. 6a, b). Moreover, iron-induced activation of p-AMPK was observed in a dose-dependent manner (FAC 10 µM and 100 µM). Taken together, we found iron may affect AMPK and subsequently promote mTORC1 activity.

## Discussion

Numerous evidences have demonstrated that iron is associated with the initiation and development of several malignant tumors [17, 19, 21]. Iron overload promotes carcinogenesis and tumorigenesis in colorectal and lung cancer [17–19]. Moreover, several studies have also demonstrated the therapeutic effects of iron chelator in lung cancer, pancreatic cancer, multiple myeloma and colorectal cancer [15, 17–19, 21, 28]. In the present study, we generated a tumor-load model by injecting SAOS-2 cell line in nude mice. After 14 days of DFO treatment, tumor volume and weight were significantly reduced. These results implied that iron maybe a promoter in osteosarcoma. Based on the epidemiological evidences that cancer patient showed with an increased body iron storage [28], so we do not set a iron-treated group in animal experiment though. To our surprise, iron chelator DFO could significantly reduce the tumor volume and weight, which implied that iron maybe a promoter in the development of osteosarcoma.

The mechanisms underlying iron and the carcinogenesis in osteosarcoma are not yet well understood. Studies have reported that intracellular iron excess resulted in the generation of ROS, degradation of P53, and the activation of gp130/STAT3 signaling pathway [17]. Iron-mediated ROS promotes Kras-driven pancreatic tumorigenesis [21]. Ferroportin-mediated ROS up-regulation promotes the proliferation of multiple myeloma [31]. Studies have also revealed that ROS can affect cell metabolism and mitochondrial function, thus influencing Warburg effect [32]. In our work, we found that mitochondrion-mediated iron accumulation played an important role in the development of osteosarcoma through ROS. Further, mitochondrion-mediated iron accumulation contributed to the proliferation, migration and invasion of osteosarcoma, as well as the Warburg effect by ROS.

Warburg effect, as one of the hallmarks of cancer, presented as a metabolic disorder. Warburg effect is associated closely with proliferation, metastasis and drug resistance of cancer cell. In our study, seahorse metabolic analysis showed a higher aerobic glycolysis and a decrease of oxygen consumption rate, a classical Warburg phenotype, which implied that iron could be a factor in this process. Several studies have demonstrated that reactive oxygen species (ROS) regulate Warburg effect directly or indirectly [21, 24–26]. In normal cells, ROS production is mainly from mitochondrion intracellular [33]. Interestingly, our work revealed that mitochondrion-mediated iron accumulation increased ROS production in both osteosarcoma cell lines, whereas knock-down of mitoferrin 1 (SLC25A37) and mitoferrin 2 (SLC25A28) decreased this phenomenon. Accordingly, our work proved that mitochondrion-mediated iron accumulation played a critical role in Warburg effect in osteosarcoma. We further explored the correlation between mitoferrin genes and Warburg key genes. Surprisingly, mitoferrin 1 (SLC25A37) had a positive correlation with HK2, GLUT1, PGK1, ENO1 and PKM, whereas mitoferrin 2 (SLC25A28) had a negative correlation with those genes. Moreover, we found that the expression of HK2 and GLUT1 was increased in iron-treated group. These results suggested that iron maybe a risk factor, which increases the expression of Warburg key genes of sarcoma patients. Given the fact that we are unable to collect osteosarcoma specimen due to the lack of such patients in our hospital, so we didn’t perform those genes expression by WB or IHC staining.

AMP-activated protein kinase (AMPK) worked as a regulator of cell metabolism and a sensor of energy, it could activate the mTOR complex 1 (mTORC1) signaling pathway when AMPK is inhibited. According to our previous results, iron may promote the proliferation of SAOS-2 and U2OS cell lines in vitro. Moreover, iron may promote the Warburg effect according to Seahorse metabolic analysis, all of which made us believe that the energy consumption of two osteosarcoma cell lines was improved significantly when iron was added into the culture medium. In addition, an improvement of aerobic glycolysis in osteosarcoma cell lines, which confirmed by Seahorse, resulting in an abundance of ATP and the essential materials of cells needed in cytoplasm, making osteosarcoma cells in a nutrition-rich condition thus rendering the inactive of AMPK as other study reported [30]. Further, since energy stress-induced activation of AMPK is also known to inhibit the activation of mTORC1, and mTORC1 is activated in the inverse pattern [34]. PI3k-AKT-mTOTC axis is an effective alternative proved by several clinical trials in the treatment of cancer [35]. Recently, study has found the inhibition of HK2 activated AMPK and thus suppressed its down-stream mTORC1 in lung cancer [34]. We further investigated the AMPK-mTORC1 signaling axis at the end of our work. The inhibition of p-AMPK and activation of its down-stream mTORC1, as indicated by the level of S6K1 (Thr389) and 4EBP1 (Thr70) phosphorylation, was observed when osteosarcoma cells treated with iron. Taken together, we demonstrated that iron could affect osteosarcoma cells metabolism by ROS and AMPK signaling pathway. These findings, in turn, suggested that iron chelator could be an effective therapeutic alternative in osteosarcoma.

## Conclusions

Our results highlighted the iron-induced carcinogenesis of osteosarcoma, and proved that mitochondrion-mediated iron accumulation promotes carcinogenesis and Warburg effect of osteosarcoma through reactive oxygen species. These findings may also provide some clues to elucidating the underlying mechanism of iron-dependent disease, especially in osteosarcoma. These findings in turn suggested that iron chelator maybe a therapeutic alternative in osteosarcoma.

## Supplementary information

**Additional file 1: Table S1.** Primers used in the real-time PCR. **Table S2.** Primers used in shRNA

**Additional file 2: Figure** **S1.** Depletion of mitoferrin 1 (SLC25A37) and mitoferrin 2 (SLC25A28) were tested by Western blot and qPCR. a. Western blot showed the expression of mitoferrin 1 (SLC25A37) and mitoferrin 2 (SLC25A28) on protein level. b. qPCR results of depletion of mitoferrin 1 (SLC25A37) and mitoferrin 2 (SLC25A28) by shRNA. (*p < 0.05, **p < 0.01, ns: no significant, mean ± SD, n = 3)

## Data Availability

The datasets used and/or analyzed during the current study are available from the corresponding author upon reasonable request.

## Abbreviations

DMT1: Divalent metal transporter 1
FPN1: Ferroportin 1
ROS: Reactive oxygen species
FAC: Ferric ammonium citrate
DFO: Deferoxamine
DCFH-DA: Dichlorodihydrofluorescein diacetate
OCR: Oxygen consumption rate
ECAR: Extracellular acid rate
AMPK: AMP-activated protein kinase
mTORC1: mTOR complex 1
