# Supplementary material for: Mitochondrion-mediated iron accumulation promotes carcinogenesis and Warburg effect through reactive oxygen species in osteosarcoma
Source: Cancer Cell Int. 2020 Aug 18;20:399. doi: 10.1186/s12935-020-01494-3 (PMC7437012; doi:10.1186/s12935-020-01494-3)
Supplement: Supplementary file 1 — Additional file 1: Table S1. Primers used in the real-time PCR. Table S2. Primers used in shRNA [file 12935_2020_1494_MOESM1_ESM.docx]

Table S1. Primers used in the real-time PCR

| Genes | Forward (5′-3′) | Reverse (5′-3′) |
| --- | --- | --- |
| Actin | GGGACCTGACTGACTACCTC | TCATACTCCTGCTTGCTGAT |
| SLC25A37 | GATGGGGACAGCCGAGATG | ACCGGGTACATGACCGAGT |
| SLC25A28 | CTTTCCCAACTCTTTGGGC | CTCCAAACCATGCTGAGAC |

Table S2. Primers used in shRNA

| Genes | Sequence |
| --- | --- |
| Human shSLC25A37 | CCGGAGGCGTCAACGTCATGATCATCTCGAGATGATCATGACGTTGACGCCTTTTTTG |
| Human shSLC25A28 | CCGGCTTGGCTTTGAACTCACACATCTCGAGATGTGTGAGTTCAAAGCCAAGTTTTTG |
