# Supplementary figures and images for: Mitochondrion-mediated iron accumulation promotes carcinogenesis and Warburg effect through reactive oxygen species in osteosarcoma
Source: Cancer Cell Int. 2020 Aug 18;20:399. doi: 10.1186/s12935-020-01494-3 (PMC7437012; doi:10.1186/s12935-020-01494-3)

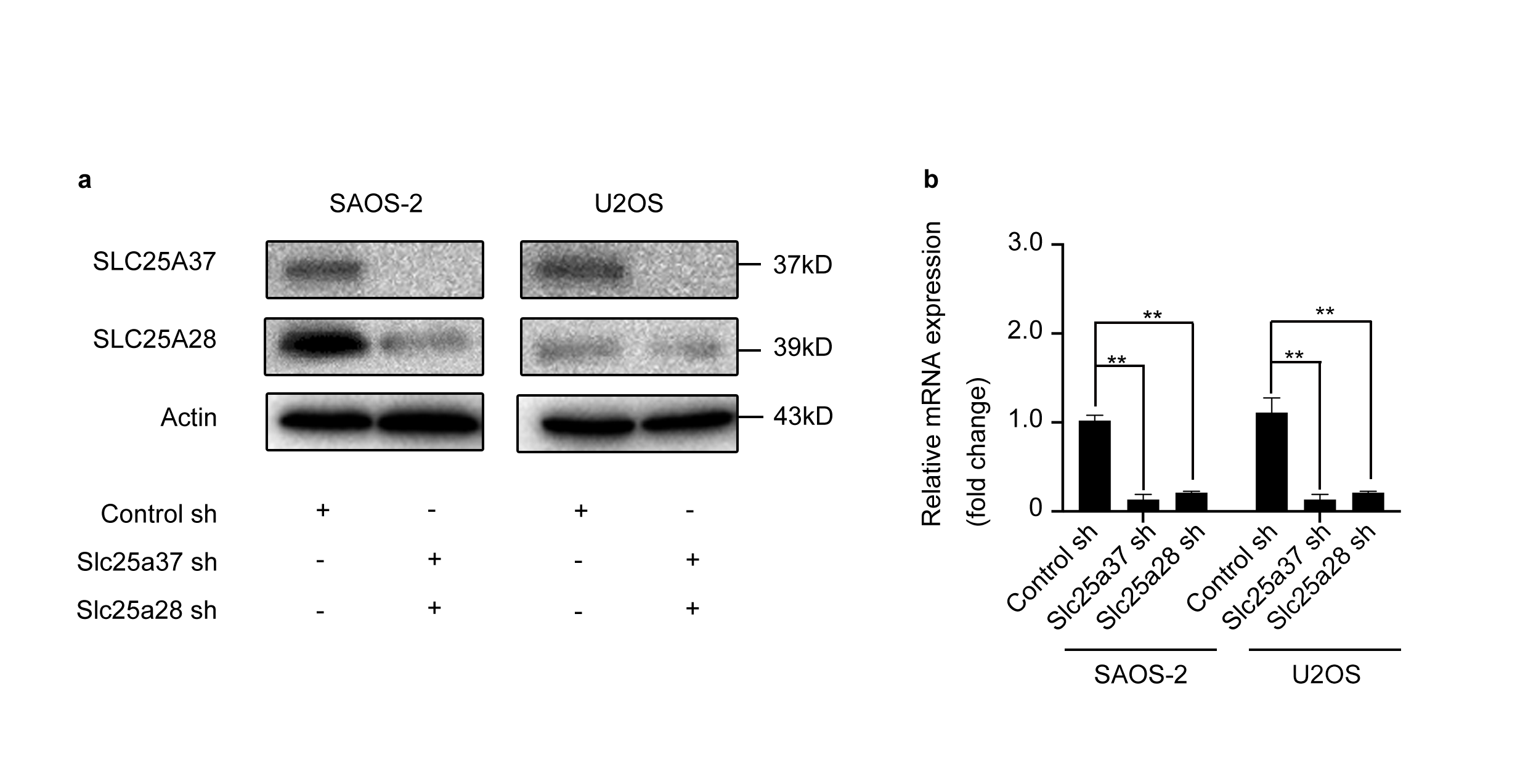

Supplement: Supplementary file 2 — Additional file 2: Figure S1. Depletion of mitoferrin 1 (SLC25A37) and mitoferrin 2 (SLC25A28) were tested by Western blot and qPCR. a. Western blot showed the expression of mitoferrin 1 (SLC25A37) and mitoferrin 2 (SLC25A28) on protein level. b. qPCR results of depletion of mitoferrin 1 (SLC25A37) and mitoferrin 2 (SLC25A28) by shRNA. (*p < 0.05, **p < 0.01, ns: no significant, mean ± SD, n = 3) [file 12935_2020_1494_MOESM2_ESM.tif]
